# *trans*-Bis[(1-ammonio­pentane-1,1-di­yl)diphospho­nato-κ^2^ *O*,*O*′]diaqua­copper(II)

**DOI:** 10.1107/S1600536810045216

**Published:** 2010-11-10

**Authors:** Natalia V. Tsaryk, Anatolij V. Dudko, Alexandra N. Kozachkova, Vladimir V. Bon, Vasily I. Pekhnyo

**Affiliations:** a Institute of General and Inorganic Chemistry, NAS Ukraine, prosp. Palladina 32/34, Kyiv 03680, Ukraine

## Abstract

In the title compound, [Cu(C_5_H_14_NO_6_P_2_)_2_(H_2_O)_2_], the Cu^II^ atom occupies a special position on an inversion centre. It exhibits a distorted octa­hedral coordination environment consisting of two *O*,*O*′-bidentate (1-ammonio­pentane-1,1-di­yl)diphospho­nate anions in the equatorial plane and two *trans* water mol­ecules located in axial positions. The ligand mol­ecules are coordinated to the Cu^II^ atom in their zwitterionic form *via* two O atoms from different phospho­nate groups, creating two six–membered chelate rings with a screw-boat conformation. The CuO_6_ coordination polyhedron is strongly elongated in the axial direction with 0.6 Å longer bonds than those in the equatorial plane. Intra­molecular N—H⋯O hydrogen bonding helps to stabilize the mol­ecular configuration. The presence of supra­molecular —PO(OH)⋯O(OH)P— units parallel to (100) and other O—H⋯O and N—H⋯O hydrogen bonds establish the three-dimensional set-up.

## Related literature

For general background to organic diphospho­nic acids and their metal complexes, see: Eberhardt *et al.* (2005 ▶); Matczak-Jon & Videnova-Adrabinska (2005 ▶). For related structures, see: Sergienko *et al.* (1997 ▶, 1999 ▶).
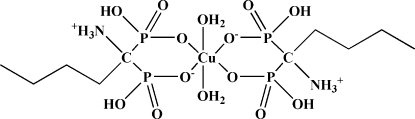


## Experimental

### 

#### Crystal data

[Cu(C_5_H_14_NO_6_P_2_)_2_(H_2_O)_2_]
*M* *_r_* = 591.80
Triclinic, 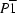

*a* = 5.5629 (1) Å
*b* = 10.0236 (2) Å
*c* = 10.5237 (2) Å
α = 69.315 (1)°
β = 86.666 (1)°
γ = 88.398 (1)°
*V* = 548.03 (2) Å^3^
*Z* = 1
Mo *K*α radiation
μ = 1.36 mm^−1^
*T* = 173 K
0.35 × 0.15 × 0.08 mm

#### Data collection

Bruker APEXII CCD diffractometer
Absorption correction: multi-scan (*SADABS*; Bruker, 2005 ▶) *T*_min_ = 0.648, *T*_max_ = 0.899
5322 measured reflections
2277 independent reflections
2104 reflections with *I* > 2σ(*I*)
*R*_int_ = 0.019

#### Refinement

*R*[*F* ^2^ > 2σ(*F* ^2^)] = 0.023
*wR*(*F* ^2^) = 0.064
*S* = 1.07
2277 reflections
168 parameters
4 restraints
H atoms treated by a mixture of independent and constrained refinement
Δρ_max_ = 0.43 e Å^−3^
Δρ_min_ = −0.36 e Å^−3^

### 

Data collection: *APEX2* (Bruker, 2005 ▶); cell refinement: *SAINT* (Bruker, 2005 ▶); data reduction: *SAINT*; program(s) used to solve structure: *SHELXS97* (Sheldrick, 2008 ▶); program(s) used to refine structure: *SHELXL97* (Sheldrick, 2008 ▶); molecular graphics: *DIAMOND* (Brandenburg & Putz, 2010 ▶); software used to prepare material for publication: *publCIF* (Westrip, 2010 ▶).

## Supplementary Material

Crystal structure: contains datablocks I, global. DOI: 10.1107/S1600536810045216/wm2420sup1.cif

Structure factors: contains datablocks I. DOI: 10.1107/S1600536810045216/wm2420Isup2.hkl

Additional supplementary materials:  crystallographic information; 3D view; checkCIF report

## Figures and Tables

**Table 1:** Selected bond lengths (Å)

Cu1—O4	1.9381 (12)
Cu1—O1	1.9524 (12)
Cu1—O7	2.5666 (15)

**Table 2:** Hydrogen-bond geometry (Å, °)

*D*—H⋯*A*	*D*—H	H⋯*A*	*D*⋯*A*	*D*—H⋯*A*
N1—H11*N*⋯O3^i^	0.91 (2)	1.98 (3)	2.849 (2)	158 (2)
N1—H12*N*⋯O7^ii^	0.88 (2)	2.08 (3)	2.945 (2)	167 (2)
N1—H13*N*⋯O5^i^	0.89 (3)	1.99 (3)	2.849 (2)	162 (2)
O2—H2*O*⋯O3^iii^	0.79 (2)	1.79 (2)	2.5741 (18)	178 (3)
O6—H6*O*⋯O5^iv^	0.79 (2)	1.80 (2)	2.5848 (18)	176 (3)
O7—H71*O*⋯O4^v^	0.79 (2)	2.04 (2)	2.8071 (19)	165 (3)
O7—H72*O*⋯O2^ii^	0.79 (2)	2.56 (3)	3.010 (2)	118 (3)

Symmetry codes: (i) 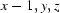; (ii) 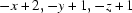; (iii) 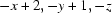; (iv) 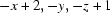; (v) 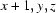.

**Fig. 1.**
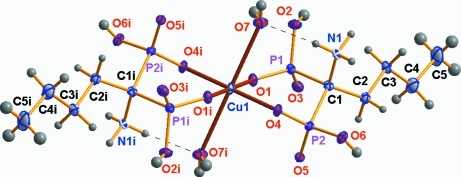
The molecular configuration of the title compound. Displacement ellipsoids are drawn at the 50% probability level. The intramolecular N—H···O hydrogen bond is emphasized by dotted lines.

**Fig. 2.**
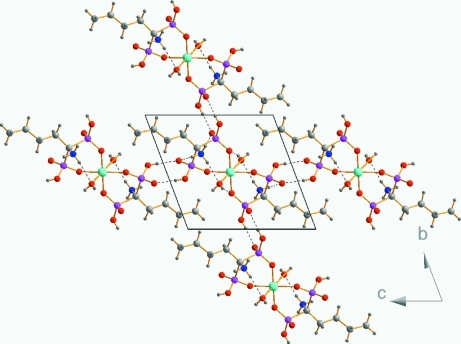
The packing diagram of the title compound viewed down the a axis. Dashed lines indicate hydrogen bonds.

## supplementary crystallographic information

### Comment

The investigation of organic diphosphonic acids and their metal complexes attracts constant interest of chemists because of their potential applications as drugs preventing calcification and inhibiting bone resorption (Matczak-Jon & Videnova-Adrabinska, 2005). Some transition metal diphosphonates can improve fixation of cementless metal implants by enhancing the extent of osteointegration (Eberhardt *et al.*, 2005). Therefore, a detailed structural investigation of diphosphonates may help to better understand their structure-property correlations.

Several structures of copper diphosphonates have been published earlier (Sergienko *et al.*, 1997, 1999). The present paper reports the structure of the first complex compound with (1-ammoniopentane-1,1-diyl)diphosphonic acid.

The asymmetric unit of title compound contains one half of the molecule. The Cu^II^ atom occupies a special position on a crystallographic inversion centre, which generates another half of the molecule (Fig. 1). The central Cu^II^ atom exhibits a distorted octahedral coordination geometry consisting of two *O,O'*-bidentantely coordinating ligand molecules in the equatorial plane and two *trans* water molecules, located in the axial positions. The ligand molecules are coordinated to Cu^II^ in their zwitterionic form *via* two O atoms from different phosphonate groups creating two six-membered chelate metalla rings with a screw-boat conformation. The CuO_6_ coordination polyhedron is strongly elongated in the axial direction: The Cu1—O7 bond is ~ 0.6 Å longer than the Cu1—O1 and Cu1—O4 bonds (Table 1), which is characteristic for Jahn-Teller distorted Cu^II^ complexes with an octahedral coordination (Sergienko *et al.*, 1997). The values of the equatorial O—Cu—O angles are in the range of 80.05 (5)–99.95 (5)°, indicating a significiant deviation from the ideal values. This can be explained by the presence of a strong intramolecular hydrogen bond N1—H12···O7 (Fig. 1, Table 2), which partially influences the configuration of the molecule. The crystal structure of title compound contains supramolecular units —PO(OH)···O(OH)P— parallel to (100) that, together with strong O—H···O and N—H···O hydrogen bonds, create a three-dimensional structure (Fig. 2, Table 2).

### Experimental

Light blue crystals of the title compound were obtained from the mixture of CuSO_4_^.^5H_2_O (0.2 mmol, 0.04995 g) and 1-aminopentane-1,1-diyldiphosphonic acid (0.4 mmol, 0.09885 g) in 5 ml of H_2_O, adjusted to pH ~ 4 with 0.25*M* NaOH. The combined solution was stored in a dark place for slow evaporation. After 20 days of staying, suitable crystals for X-ray data collection were obtained.

### Refinement

H atoms bonded to O and N atoms were located in a difference map and refined with distance restraint of 0.82 (2) Å for OH and without any restraints for NH. Other H atoms, which are bonded to C atoms, were positioned geometrically regarding to hybridization and refined using a riding model with C—H = 0.98 Å for CH_3_ [*U*_iso_(H) = 1.5Ueq(C)] and C—H = 0.99 Å for CH_2_ [*U*_iso_(H) = 1.2Ueq(C)].

### Crystal data

**Table:**

[Cu(C_5_H_14_NO_6_P_2_)_2_(H_2_O)_2_]	*Z* = 1
*M**_r_* = 591.80	*F*(000) = 307
Triclinic, *P*1	*D*_x_ = 1.793 Mg m^−^^3^
Hall symbol: -P 1	Mo *K*α radiation, λ = 0.71073 Å
*a* = 5.5629 (1) Å	Cell parameters from 3621 reflections
*b* = 10.0236 (2) Å	θ = 2.4–26.6°
*c* = 10.5237 (2) Å	µ = 1.36 mm^−^^1^
α = 69.315 (1)°	*T* = 173 K
β = 86.666 (1)°	Rod, light blue
γ = 88.398 (1)°	0.35 × 0.15 × 0.08 mm
*V* = 548.03 (2) Å^3^	

### Data collection

**Table:**

Bruker APEXII CCD diffractometer	2277 independent reflections
Radiation source: fine-focus sealed tube	2104 reflections with *I* > 2σ(*I*)
graphite	*R*_int_ = 0.019
φ and ω scans	θ_max_ = 26.7°, θ_min_ = 2.1°
Absorption correction: multi-scan (*SADABS*; Bruker, 2005)	*h* = −7→6
*T*_min_ = 0.648, *T*_max_ = 0.899	*k* = −12→12
5322 measured reflections	*l* = −13→13

### Refinement

**Table:**

Refinement on *F*^2^	Primary atom site location: structure-invariant direct methods
Least-squares matrix: full	Secondary atom site location: difference Fourier map
*R*[*F*^2^ > 2σ(*F*^2^)] = 0.023	Hydrogen site location: inferred from neighbouring sites
*wR*(*F*^2^) = 0.064	H atoms treated by a mixture of independent and constrained refinement
*S* = 1.07	*w* = 1/[σ^2^(*F*_o_^2^) + (0.0298*P*)^2^ + 0.4491*P*] where *P* = (*F*_o_^2^ + 2*F*_c_^2^)/3
2277 reflections	(Δ/σ)_max_ < 0.001
168 parameters	Δρ_max_ = 0.43 e Å^−^^3^
4 restraints	Δρ_min_ = −0.36 e Å^−^^3^

### Special details

**Table:**

Geometry. All e.s.d.'s (except the e.s.d. in the dihedral angle between two l.s. planes) are estimated using the full covariance matrix. The cell e.s.d.'s are taken into account individually in the estimation of e.s.d.'s in distances, angles and torsion angles; correlations between e.s.d.'s in cell parameters are only used when they are defined by crystal symmetry. An approximate (isotropic) treatment of cell e.s.d.'s is used for estimating e.s.d.'s involving l.s. planes.
Refinement. Refinement of *F*^2^ against ALL reflections. The weighted *R*-factor *wR* and goodness of fit *S* are based on *F*^2^, conventional *R*-factors *R* are based on *F*, with *F* set to zero for negative *F*^2^. The threshold expression of *F*^2^ > σ(*F*^2^) is used only for calculating *R*-factors(gt) *etc*. and is not relevant to the choice of reflections for refinement. *R*-factors based on *F*^2^ are statistically about twice as large as those based on *F*, and *R*- factors based on ALL data will be even larger.

### Fractional atomic coordinates and isotropic or equivalent isotropic displacement parameters (Å^2^)

**Table:**

	*x*	*y*	*z*	*U*_iso_*/*U*_eq_	
Cu1	1.0000	0.5000	0.5000	0.01029 (10)	
P1	0.98580 (8)	0.44423 (5)	0.21486 (4)	0.00887 (11)	
P2	0.90052 (8)	0.20736 (5)	0.48297 (5)	0.00893 (11)	
N1	0.5396 (3)	0.34163 (17)	0.32262 (17)	0.0112 (3)	
H11N	0.458 (4)	0.367 (2)	0.245 (3)	0.017*	
H12N	0.539 (4)	0.416 (3)	0.348 (2)	0.017*	
H13N	0.444 (4)	0.274 (3)	0.380 (2)	0.017*	
O1	1.0859 (2)	0.49686 (13)	0.31854 (12)	0.0116 (3)	
O2	0.8085 (2)	0.55876 (14)	0.12618 (14)	0.0136 (3)	
H2O	0.813 (5)	0.573 (3)	0.0476 (18)	0.040 (8)*	
O3	1.1764 (2)	0.40075 (14)	0.12969 (13)	0.0120 (3)	
O4	0.8517 (2)	0.31422 (13)	0.55360 (13)	0.0114 (3)	
O5	1.1555 (2)	0.15980 (13)	0.46941 (13)	0.0119 (3)	
O6	0.7259 (2)	0.08082 (14)	0.55649 (14)	0.0131 (3)	
H6O	0.767 (5)	0.009 (2)	0.546 (3)	0.033 (8)*	
C1	0.7927 (3)	0.28852 (18)	0.31004 (18)	0.0098 (3)	
C2	0.7970 (3)	0.17564 (19)	0.24218 (19)	0.0136 (4)	
H2A	0.9659	0.1442	0.2349	0.016*	
H2B	0.7053	0.0919	0.3029	0.016*	
C3	0.6952 (4)	0.2217 (2)	0.10125 (19)	0.0156 (4)	
H3A	0.5172	0.2165	0.1103	0.019*	
H3B	0.7404	0.3217	0.0488	0.019*	
C4	0.7925 (4)	0.1255 (2)	0.0260 (2)	0.0231 (4)	
H4A	0.7598	0.0250	0.0831	0.028*	
H4B	0.9693	0.1369	0.0113	0.028*	
C5	0.6820 (5)	0.1581 (3)	−0.1105 (2)	0.0337 (6)	
H5A	0.5063	0.1523	−0.0973	0.050*	
H5B	0.7410	0.0886	−0.1515	0.050*	
H5C	0.7275	0.2543	−0.1709	0.050*	
O7	1.3880 (3)	0.39603 (15)	0.61934 (15)	0.0182 (3)	
H71O	1.511 (4)	0.358 (3)	0.610 (3)	0.034 (8)*	
H72O	1.335 (5)	0.342 (3)	0.689 (2)	0.045 (9)*	

### Atomic displacement parameters (Å^2^)

**Table:**

	*U*^11^	*U*^22^	*U*^33^	*U*^12^	*U*^13^	*U*^23^
Cu1	0.01286 (17)	0.01044 (16)	0.00899 (16)	−0.00195 (11)	0.00097 (12)	−0.00526 (12)
P1	0.0090 (2)	0.0103 (2)	0.0078 (2)	−0.00018 (16)	−0.00004 (17)	−0.00388 (17)
P2	0.0094 (2)	0.0088 (2)	0.0089 (2)	−0.00008 (16)	−0.00038 (17)	−0.00345 (17)
N1	0.0092 (8)	0.0124 (8)	0.0121 (8)	−0.0011 (6)	−0.0007 (6)	−0.0045 (7)
O1	0.0125 (6)	0.0135 (6)	0.0099 (6)	−0.0031 (5)	0.0013 (5)	−0.0055 (5)
O2	0.0160 (7)	0.0145 (6)	0.0094 (7)	0.0037 (5)	−0.0007 (5)	−0.0035 (5)
O3	0.0106 (6)	0.0161 (6)	0.0098 (6)	0.0010 (5)	0.0003 (5)	−0.0057 (5)
O4	0.0140 (6)	0.0109 (6)	0.0104 (6)	−0.0023 (5)	0.0013 (5)	−0.0050 (5)
O5	0.0105 (6)	0.0105 (6)	0.0151 (6)	0.0001 (5)	−0.0013 (5)	−0.0050 (5)
O6	0.0140 (7)	0.0097 (6)	0.0160 (7)	−0.0013 (5)	0.0030 (5)	−0.0053 (5)
C1	0.0082 (8)	0.0114 (8)	0.0109 (8)	0.0000 (6)	0.0000 (7)	−0.0051 (7)
C2	0.0167 (9)	0.0122 (9)	0.0138 (9)	−0.0007 (7)	−0.0015 (7)	−0.0069 (7)
C3	0.0182 (10)	0.0168 (9)	0.0138 (9)	0.0000 (7)	−0.0043 (7)	−0.0075 (8)
C4	0.0331 (12)	0.0222 (10)	0.0175 (10)	0.0016 (9)	−0.0021 (9)	−0.0117 (9)
C5	0.0554 (17)	0.0308 (12)	0.0196 (11)	−0.0073 (11)	−0.0052 (11)	−0.0137 (10)
O7	0.0157 (7)	0.0187 (7)	0.0184 (8)	0.0027 (6)	0.0016 (6)	−0.0050 (6)

### Geometric parameters (Å, °)

**Table:**

Cu1—O4	1.9381 (12)	O2—H2O	0.787 (17)
Cu1—O4^i^	1.9381 (12)	O6—H6O	0.791 (17)
Cu1—O1	1.9524 (12)	C1—C2	1.536 (2)
Cu1—O1^i^	1.9524 (12)	C2—C3	1.527 (3)
Cu1—O7	2.5666 (15)	C2—H2A	0.9900
Cu1—O7^i^	2.5666 (15)	C2—H2B	0.9900
P1—O3	1.5023 (13)	C3—C4	1.520 (3)
P1—O1	1.5075 (13)	C3—H3A	0.9900
P1—O2	1.5649 (13)	C3—H3B	0.9900
P1—C1	1.8594 (18)	C4—C5	1.520 (3)
P2—O5	1.4986 (13)	C4—H4A	0.9900
P2—O4	1.5153 (13)	C4—H4B	0.9900
P2—O6	1.5618 (14)	C5—H5A	0.9800
P2—C1	1.8404 (18)	C5—H5B	0.9800
N1—C1	1.507 (2)	C5—H5C	0.9800
N1—H11N	0.91 (2)	O7—H71O	0.791 (17)
N1—H12N	0.88 (2)	O7—H72O	0.786 (17)
N1—H13N	0.89 (3)		
			
O4—Cu1—O4^i^	180.0	N1—C1—P2	107.55 (12)
O4—Cu1—O1	91.21 (5)	C2—C1—P2	109.19 (12)
O4^i^—Cu1—O1	88.79 (5)	N1—C1—P1	108.41 (12)
O4—Cu1—O1^i^	88.79 (5)	C2—C1—P1	112.60 (12)
O4^i^—Cu1—O1^i^	91.21 (5)	P2—C1—P1	108.34 (9)
O1—Cu1—O1^i^	180.0	C3—C2—C1	116.32 (15)
O4—Cu1—O7	92.80 (5)	C3—C2—H2A	108.2
O4^i^—Cu1—O7	87.20 (5)	C1—C2—H2A	108.2
O1—Cu1—O7	99.95 (5)	C3—C2—H2B	108.2
O1^i^—Cu1—O7	80.05 (5)	C1—C2—H2B	108.2
O3—P1—O1	113.56 (7)	H2A—C2—H2B	107.4
O3—P1—O2	112.23 (7)	C4—C3—C2	110.18 (16)
O1—P1—O2	109.39 (7)	C4—C3—H3A	109.6
O3—P1—C1	109.48 (8)	C2—C3—H3A	109.6
O1—P1—C1	107.00 (8)	C4—C3—H3B	109.6
O2—P1—C1	104.67 (8)	C2—C3—H3B	109.6
O5—P2—O4	118.15 (7)	H3A—C3—H3B	108.1
O5—P2—O6	113.02 (7)	C3—C4—C5	112.75 (18)
O4—P2—O6	105.54 (7)	C3—C4—H4A	109.0
O5—P2—C1	107.25 (8)	C5—C4—H4A	109.0
O4—P2—C1	106.99 (8)	C3—C4—H4B	109.0
O6—P2—C1	105.01 (8)	C5—C4—H4B	109.0
C1—N1—H11N	114.7 (14)	H4A—C4—H4B	107.8
C1—N1—H12N	110.8 (15)	C4—C5—H5A	109.5
H11N—N1—H12N	107 (2)	C4—C5—H5B	109.5
C1—N1—H13N	112.7 (15)	H5A—C5—H5B	109.5
H11N—N1—H13N	101 (2)	C4—C5—H5C	109.5
H12N—N1—H13N	109 (2)	H5A—C5—H5C	109.5
P1—O1—Cu1	139.17 (8)	H5B—C5—H5C	109.5
P1—O2—H2O	118 (2)	Cu1—O7—H71O	142 (2)
P2—O4—Cu1	124.94 (8)	Cu1—O7—H72O	101 (2)
P2—O6—H6O	113 (2)	H71O—O7—H72O	101 (3)
N1—C1—C2	110.60 (15)		
			
O3—P1—O1—Cu1	−148.54 (11)	O5—P2—C1—P1	−60.90 (10)
O2—P1—O1—Cu1	85.23 (13)	O4—P2—C1—P1	66.79 (10)
C1—P1—O1—Cu1	−27.63 (14)	O6—P2—C1—P1	178.62 (8)
O4—Cu1—O1—P1	35.20 (12)	O3—P1—C1—N1	−146.05 (11)
O4^i^—Cu1—O1—P1	−144.80 (12)	O1—P1—C1—N1	90.47 (12)
O7—Cu1—O1—P1	128.25 (12)	O2—P1—C1—N1	−25.56 (13)
O5—P2—O4—Cu1	56.52 (11)	O3—P1—C1—C2	−23.36 (14)
O6—P2—O4—Cu1	−175.96 (8)	O1—P1—C1—C2	−146.83 (12)
C1—P2—O4—Cu1	−64.49 (11)	O2—P1—C1—C2	97.14 (13)
O1—Cu1—O4—P2	19.60 (9)	O3—P1—C1—P2	97.52 (9)
O1^i^—Cu1—O4—P2	−160.40 (9)	O1—P1—C1—P2	−25.95 (10)
O7—Cu1—O4—P2	−80.42 (9)	O2—P1—C1—P2	−141.98 (8)
O5—P2—C1—N1	−177.89 (11)	N1—C1—C2—C3	57.4 (2)
O4—P2—C1—N1	−50.20 (13)	P2—C1—C2—C3	175.60 (14)
O6—P2—C1—N1	61.64 (13)	P1—C1—C2—C3	−64.01 (19)
O5—P2—C1—C2	62.07 (14)	C1—C2—C3—C4	158.78 (17)
O4—P2—C1—C2	−170.25 (12)	C2—C3—C4—C5	175.28 (19)
O6—P2—C1—C2	−58.41 (14)		

Symmetry codes: (i) −*x*+2, −*y*+1, −*z*+1.

### Hydrogen-bond geometry (Å, °)

**Table:**

*D*—H···*A*	*D*—H	H···*A*	*D*···*A*	*D*—H···*A*
N1—H11N···O3^ii^	0.91 (2)	1.98 (3)	2.849 (2)	158 (2)
N1—H12N···O7^i^	0.88 (2)	2.08 (3)	2.945 (2)	167 (2)
N1—H13N···O5^ii^	0.89 (3)	1.99 (3)	2.849 (2)	162 (2)
O2—H2O···O3^iii^	0.79 (2)	1.79 (2)	2.5741 (18)	178 (3)
O6—H6O···O5^iv^	0.79 (2)	1.80 (2)	2.5848 (18)	176 (3)
O7—H71O···O4^v^	0.79 (2)	2.04 (2)	2.8071 (19)	165 (3)
O7—H72O···O2^i^	0.79 (2)	2.56 (3)	3.010 (2)	118 (3)

Symmetry codes: (ii) *x*−1, *y*, *z*; (i) −*x*+2, −*y*+1, −*z*+1; (iii) −*x*+2, −*y*+1, −*z*; (iv) −*x*+2, −*y*, −*z*+1; (v) *x*+1, *y*, *z*.
